# Editorial: Body fluid biomarkers in neurodegenerative studies: Novel insights into pathophysiology to support clinical practice and drug development

**DOI:** 10.3389/fneur.2023.1103116

**Published:** 2023-01-17

**Authors:** Anastasia Bougea, Per Svenningsson, Ioanna Markaki, Abdul Hye, Stefania Mondello

**Affiliations:** ^1^First Department of Neurology, Eginition Hospital, National and Kapodistrian University of Athens, Athens, Greece; ^2^Department of Clinical Neuroscience, Karolinska Institutet, Stockholm, Sweden; ^3^Basic and Clinical Neuroscience, King's College London, London, United Kingdom; ^4^Center of Neurology, Academic Specialist Center, Stockholm, Sweden; ^5^Department of Old Age Psychiatry, Institute of Psychiatry, Psychology, and Neuroscience, King's College London, London, United Kingdom; ^6^Department of Biomedical and Dental Sciences and Morphofunctional Imaging University of Messina, Messina, Italy

**Keywords:** biomarkers, neurodegeneration, pathophysiology, Alzheimer's disease (AD), Parkinson's disease (PD)

## Introduction

Neurodegenerative diseases are a heterogeneous group of disorders characterized by progressive neuronal loss in the central nervous system (1). These include, among others, Alzheimer's Disease (AD), Parkinson's disease (PD), and Huntington's disease (HD). Although monoclonal antibodies have become the first AD treatment to potentially delay the decline caused by dementia, there are no obviously established effective treatments for other neurodegenerative diseases to reverse the neurodegenerative mechanisms that cause disease progression (2). Currently available therapies only stabilize/slow disease-related symptoms (1). Sensitive, easy-to-measure biomarkers reflecting brain pathophysiology and capable of improving accurate patient phenotyping and prognosis would represent a key advance for informing treatment decisions and drug development, as well as for therapy monitoring. To date, a myriad of studies exploring protein biomarkers (e.g., α-synuclein [α-syn], amyloid-β42 [Aβ42], total tau [t-tau], phosphorylated-181-tau [P-tau181], and neurofilament light chain [NfL]) in the cerebrospinal fluid (CSF) and in the blood of patients with neurodegenerative diseases have been conducted (3–8). Nonetheless, further research is required to gain deeper insights into mechanisms underpinning biomarker release and increase, as well as their links with disease pathogenesis.

This Research Topic of Frontiers in Neurology entitled “*Body Fluid Biomarkers in Neurodegenerative Studies: Novel Insights into Pathophysiology to Support Clinical Practice and Drug Development*” includes articles investigating the role of biomarkers in disease pathogenesis and, thereby, their potential for monitoring neurodegenerative processes and designing mechanism-based therapies.

## Summary of articles

Neurodegeneration-related CSF biomarkers, such as α-syn, Aβ42, t-tau, and p-tau, have previously been studied in PD dementia (3). However, studies on blood-based biomarkers in PD are limited. In a pilot study on 22 PD and 10 healthy subjects, Schirinz et al. measured plasma and CSF levels of tau and amyloid-beta peptides and showed that in the PD group, inter-fluid (serum-CSF) levels of tau and Aβ42 correlated strongly. No correlation between the two components was found in the control group. These data, although preliminary, suggest that increased blood levels of these markers may be a reliable indicator of CSF levels in PD patients.

Soluble triggering receptor expressed on myeloid cells 2 (sTrem2) in CSF is increasingly recognized as a reliable indicator for neuroinflammation, while iron accumulation is found in several neurodegenerative conditions. In the study by Shi X. et al., CSF ferritin was significantly associated with sTrem2 in the AD continuum. These findings support further evaluation of CSF ferritin as a biomarker of the Trem2-mediated microglial function.

Using ultrasensitive single-molecule array assays (Simoa) it is possible to evaluate blood glial fibrillary acidic protein (GFAP) as a biomarker for HD-related astroglial activation. In a retrospective study of 57 HD-mutation carriers (15 premanifest HD, preHD, and 42 manifest HD) and 26 healthy controls conducted by You et al., plasma GFAP correlated with plasma NfL, and was associated with disease burden, total motor score, and total functional capacity in the HD group. This work provides initial evidence for further studies on easily accessible, plasma biomarkers of disease progression in HD.

Aneurysmal subarachnoid hemorrhage (aSAH) is a life-threatening disease that leads to neurological deficits *via* several mechanisms, such as direct injury by the hemorrhage itself, elevated intracranial pressure, and hydrocephalus (9). In a meta-analysis of 10 studies including 4,989 patients, Shi M. et al. showed that the neutrophil-to-lymphocyte ratio (NLR) was associated with poor functional outcome and the occurrence of complications (i.e., delayed cerebral ischemia [DCI]) in patients with aSAH. These results suggest that the NLR may serve as an early blood-based prognostic marker that can identify patients with a potentially severe disease course.

Minimal hepatic encephalopathy (MHE) is a neuropsychiatric and cognitive syndrome caused by liver diseases and accompanying damage to the CNS. Yang et al. investigated peripheral butyrylcholinesterase (BuChE) activity in a rat model of MHE and found that it was significantly lower than their control counterparts. Moreover, BuChE activity in frontal lobe extracts was significantly higher in MHE than in controls. In a patient population included in the study, serum BuChe activity was lowest in patients with MHE, increased gradually in the group of patients with cirrhosis but no MHE, and was highest in healthy controls, correlating with clinical scores of cognitive function. The authors speculated that the altered activity of BuChE may contribute to cognitive impairment in patients with MHE and may be a potential biomarker for liver disease severity.

Glaucoma is a neurodegenerative disease of the visual system. In a case-control study of 118 patients with primary open-angle glaucoma and 120 healthy controls, Ma et al. showed a negative association between platelet parameters and the retinal nerve fiber layer (RNEL), ganglion cell complex (GCC) thickness, and cup/disk area ratio, evaluated by optical coherence tomography (OCT). These findings suggest that platelet activation may contribute to glaucomatous optic neuropathy.

Major depressive disorder (MDD), schizophrenia (SZ), and bipolar disorder (BD) are associated with excitotoxicity and neuroinflammatory processes that, among other factors, may accelerate normal aging mechanisms. In a follow-up cohort of patients with different mental and somatic diseases, Garés-Caballer et al. used a transdiagnostic approach to elucidate the predictive power of peripheral biomarkers for immune–inflammatory activity in relation to social and executive functioning in patients with severe mental illnesses and type 2 diabetes mellitus.

Benign paroxysmal positional vertigo (BPPV) increases the risk of dementia in the 60 years of age or older population. In their meta-analysis, Liu et al. showed that there was a higher level of otolin-1 in patients with BPPV than in healthy controls. Otolin-1 may therefore serve as a biomarker for the onset of BPPV.

Overall, the articles published in this Research Topic provide insights into biochemical markers of neurodegenerative disorders and stimulate hypothesis-driven initiatives to refine future work to generate novel data in the field.

## Author contributions

All authors listed have made a substantial, direct, and intellectual contribution to the work and approved it for publication.
